# Relevant Networks involving the p53 Signalling Pathway in Renal Cell Carcinoma

**Published:** 2011-12

**Authors:** V. Medina Villaamil, G. Aparicio Gallego, I. Santamarina Caínzos, L. Valbuena Ruvira, M. Valladares-Ayerbes, L. M. Antón Aparicio

**Affiliations:** 1 *INIBIC, CHU A Coruña. A Coruña, Spain;*; 2 *Department of Pathology. Modelo Hospital. A Coruña, Spain;*; 3 *Department of Oncology. CHU A Coruña. A Coruña, Spain;*; 4 *Department of Medicine University of A Coruña. A Coruña, Spain*

**Keywords:** glucose transporters, hypoxia, p53 pathway, protein interactions, renal cell carcinoma, relevant networks

## Abstract

**Introduction:**

Renal cell carcinoma is the most common type of kidney cancer. A better understanding of the critical pathways and interactions associated with alterations in renal function and renal tumour properties is required. Our final goal is to combine the knowledge provided by a regulatory network with experimental observations provided by the dataset.

**Methods:**

In this study, a systems biology approach was used, integrating immunohistochemistry protein expression profiles and protein interaction information with the STRING and MeV bioinformatics tools. A group consisting of 80 patients with renal cell carcinoma was studied. The expression of selected markers was assessed using tissue microarray technology on immunohistochemically stained slides. The immunohistochemical data of the molecular factors studied were analysed using a parametric statistical test, Pearson’s correlation coefficient test.

**Results:**

Bioinformatics analysis of tumour samples resulted in 2 protein networks. The first network consists of proteins involved in the angiogenesis pathway and the apoptosis suppressor, BCL2, and includes both positive and negative correlations. The second network shows a negative interaction between the p53 tumour suppressor protein and the glucose transporter type 4.

**Conclusion:**

The comprehensive pathway network will help us to realise the cooperative behaviours among pathways. Regulation of metabolic pathways is an important role of p53. The pathway involving the tumour suppressor gene p53 could regulate tumour angiogenesis. Further investigation of the proteins that interact with this pathway in this type of tumour may provide new strategies for cancer therapies to specifically inhibit the molecules that play crucial roles in tumour progression.

## INTRODUCTION

Cancer is an extraordinarily complex disease of uncontrolled cellular growth, proliferation, and metastasis beyond the original tumour. Cancer is also an integrated network of signalling pathways and chemical interactions between the cancer and its human host. Renal cell carcinoma (RCC) is the most common type of kidney cancer, and it generally follows an unpredictable disease course. In 50% of human cancers, p53 is mutated, and it has become one of the most studied molecules in science (1). While at least 58 studies have investigated the role of p53 in RCC, relatively little is known with certainty about the status of p53 in RCC, in striking contrast to some other cancers. The primary cellular function of p53 is to detect acute or chronic alterations in normal cellular physiology and, more specifically, in DNA and chromosomal integrity (2, 3). However, p53 plays a number of other key roles in development (4) and tissue differentiation (5-8) and is a negative regulator of stem cell potential, demonstrating a clear relationship with cancer development (9).

Alterations in p53 expression have been observed in approximately 6–65% of human renal cancers (10), but controversy regarding their prognostic significance remains. Immunohistochemical (IHC) overexpression of p53 appears to be accompanied by metastatic progression of the disease and poor survival of patients with RCC (11). In conventional RCC, p53 expression has been correlated with the TNM Classification of Malignant Tumours (TNM) stage and metastasis, which suggests that p53 might have an important role in the progression of RCC (11). In addition, p53 overexpression is correlated with RCC tumour subtype and grade and is frequently found in the papillary, chromophobe, and clear cell RCC subtypes, as well as in high tumour grades (12, 13).

The complex interaction between genetic and environmental factors that affect multiple cellular pathways plays a role in the pathogenesis of RCC (14). The completion of the Human Genome Project in 2003 allows the systematic characterisation of comprehensive disease-associated profiles of the whole human genome. This promotes the identification of both disease-specific and stage-specific molecular signatures and biomarkers for diagnosis and prognosis prediction and targets for drug therapy (15).

Regulatory networks in eukaryotic cells consist of even more complex interactions, because numerous post-translational interactions are involved in all critical biological functions. By examining post-translational interactions, we can potentially understand all direct or indirect interactions between proteins that occur after their formation, including post-translational changes, protein-complex interactions, competition between pathways, sequestration, release, and complex inactivation (16).

Functional partnerships between proteins are at the core of complex cellular phenotypes, and the networks formed by interacting proteins have provided researchers with crucial scaffolds for modelling, data reduction and annotation. A number of algorithms have been devised that allow de novo prediction of functional links between proteins, albeit usually with considerable rates of false positives and without providing details about the specificity and type of predicted interaction (17). Many molecular network analyses go beyond protein-by-protein analysis to shed light on a system level understanding of molecular relationships between individual proteins and networks (18, 19). The dynamic structure of the human protein interaction network has been examined to aid in predicting cancer prognosis, suggesting that changes in network modularity can be used to identify tumour phenotypes (20).

Because p53 integrates multiple signalling pathways and activates numerous genes that are essential for growth arrest and apoptosis, it is considered an important inhibitor of cell proliferation and an inducer of apoptosis. Many cancers do not have p53 mutations but show reduced p53 expression (21). Links between apoptosis gene family members and key renal growth factors such as insulin-like growth factor 1, transforming growth factor β, and epidermal growth factor have been described (22).

Motivated by the dynamic structure of the human protein interaction network and the idea that interacting proteins tend to result in similar disease phenotypes when deregulated (23), we designed the present study to understand the protein-level molecular relationship between p53 and other molecules belonging to essential pathways for growth, invasiveness and tumour aggressiveness, such as angiogenic, apoptotic and metabolic pathways. Utilising IHC analysis, we carried out *in vivo* verification of our *in silico* results in our cohort of RCC samples.

Understanding the complexity of cancer depends on the elucidation of the underlying networks in the cellular, intercellular and temporal dimensions.

## MATERIALS AND METHODS

### Case material

Clinical and pathological data were obtained from patients diagnosed with RCC and who had surgery at the Department of Urology of Modelo Hospital, A Coruña, Spain, between 1996 and 2006. The study group consisted of 80 patients whose original pathological specimens were available for evaluation. The average age of the study population was 62 years, with a sex distribution of 66% male and 34% female.

The Institutional Review Board of Modelo Hospital (A Coruña, Spain) approved the retrospective review of the medical records and the use of archived tumour specimens. Informed consent was obtained from each participant.

### TMA construction

TMAs were constructed as previously described (24). Briefly, areas containing viable tumour were marked on the paraffin wax tissue blocks. Triplicate 2 mm tissue cores were taken from different areas of the same tissue block for each case (ie three cores per case), and these cores were used to construct the TMAs using an arraying machine from Durviz Instruments (Valencia, Spain). A tissue core of normal cerebellum was also included on the arrays as a negative control. Array blocks were sectioned to produce 4 μm sections.

### Immunohistochemistry

Fifteen molecular markers were chosen for investigation in this study. The choice was made taking into account proteins that we already know actively participate in providing tumour drug resistance, proteins that are therapeutic targets and, finally, proteins that have a large involvement in the growth and development of RCC. These included markers for apoptosis (BAX, BCL2, MDM2, p53 and Survivin), metabolism (Glucose transporters 1-5) and molecules involved in the angiogenesis pathway (CA9, HIF1α, VEGFA, VEGFR2 and VHL). The working dilution for the antibody panel was determined using positive controls, as indicated in the literature. Additional sections, running in parallel to but with the omission of the primary antibody, served as negative controls.

The tissue sections were deparaffinised by incubation in xylene and rehydrated in a graded series of ethanol and water solutions. The antigen was retrieved with 0.01 M citrate buffer (pH6.0) by heating the samples in a microwave vacuum histoprocessor (2100 Retriever™, PickCell Laboratories) at a controlled final temperature of 121°C for 15 minutes. Primary antibodies were diluted in Dako antibody diluent (Dakocytomation) with background-reducing components. Primary antibodies were incubated at room temperature for 30 minutes and detected using the Dako EnVision system and diaminobenzidine according to the manufacturer’s instructions.

### Scoring

All slides were scored by the same pathologist (LV). The immunoreactivity score (IRS) was evaluated similarly to other groups by multiplying the percentage of positive cells (PP %) and the staining intensity (SI). First, the PP % was scored as 0 for <1%, 1 for 1-24%, 2 for 25-49%, 3 for 50-74%, and 4 for ≥ 75%. Second, the SI was scored as 1 for weak, 2 for medium, and 3 for intense staining.

Each slide was carefully examined at the area of the tumour that contained the greatest fraction of positively stained cancer cells.

### Data analysis and statistics

Data are expressed as the mean ± standard deviation (SD). Scored results for the triplicate cores were consolidated into one score, with higher positive staining results always superseding weaker positive, negative, or uninterpretable staining results. To analyse the potential correlation between p53 protein expression and the pathological features of the study subjects, the statistical significance of the differences discovered was evaluated at the 95% confidence level by non-parametric statistics, the Mann-Whitney U and Kruskal-Wallis tests. *P* values <0.05 were considered significant. The standard Pearson correlation values for IHC data of the molecular factors studied were calculated. All of the statistical analyses were performed using commercially available software (SPSS 17.0 for Windows).

### Bioinformatics

Protein-protein interactions were obtained from the STRING (Search Tool for the Retrieval of Interacting Genes/Proteins) database (17), which contains known and predicted physical and functional protein-protein interactions. STRING was used in protein mode. The number of associations stored in STRING was shown separately for each data source and confidence range (low: scores <0.4, medium: scores from 0.4 to 0.7, and high: scores >0.7). To verify these predicted interactions in our samples and find relevant networks, we used the Multi Experiment Viewer 4.6.2 (MeV), an open-source genomic analysis software package created by the MeV Development Team and part of the TM4 Software Suite (25).

## RESULTS

### Case series description

The patient cohort included 80 patients treated with a partial or radical nephrectomy for RCC, including chromophobe, papillary and clear cell variants, between 1996 and 2006. The patients consisted of 53 men and 27 women, ranging in age from 34 to 87 years old, with a mean age of 64. There were 57 clear cell type RCC (cRCC), 15 chromophobe RCC (chRCC), 6 papillary RCC (pRCC), and 2 samples studied as histological type not determined. Regarding location, 50.6% had a right-sided tumour location, and 49.4% had left-sided tumour location. Renal pelvic invasion was present in 11% of tumours and absent in 82.5%. Only 2.5% of the cases studied showed invasion of the lymphatic vessels and the remaining 97.5% showed no lymphatic invasion. We found renal capsule rupture in 15% of tumours (n=80). Only one case showed invasion of the veins, and 98.8% were negative for this pathological parameter. Renal hilar invasion occurred in 7.3% of cases. Tumour size ranged from 2-140 cm in length. The highest level of p53 expression was observed in the papillary RCC histological type, with 2 as the highest score, indicating that a small number of cells were stained with this antibody. Regarding the degree of tissue differentiation (the Fuhrman Grade), we found that p53 expression had a significant correlation with cases identified as well differentiated and in which a Grade I p53 nuclear reaction was detected (*p*=0.031). The nuclear reaction for p53 was visualised almost exclusively in those cases with renal pelvis invasion, although this was not a statistically significant correlation (*p*=0.630). We could not detect differences in p53 expression among cases with or without ruptured capsules (*p*=0.568). Regarding tumour staging, we found that p53 was expressed more frequently in tumours diagnosed as T1-2 and in the N1-0 (*p*=0.078 and *p*=0.499, respectively).

### Identification of p53 interacting proteins

**Figure 1:**
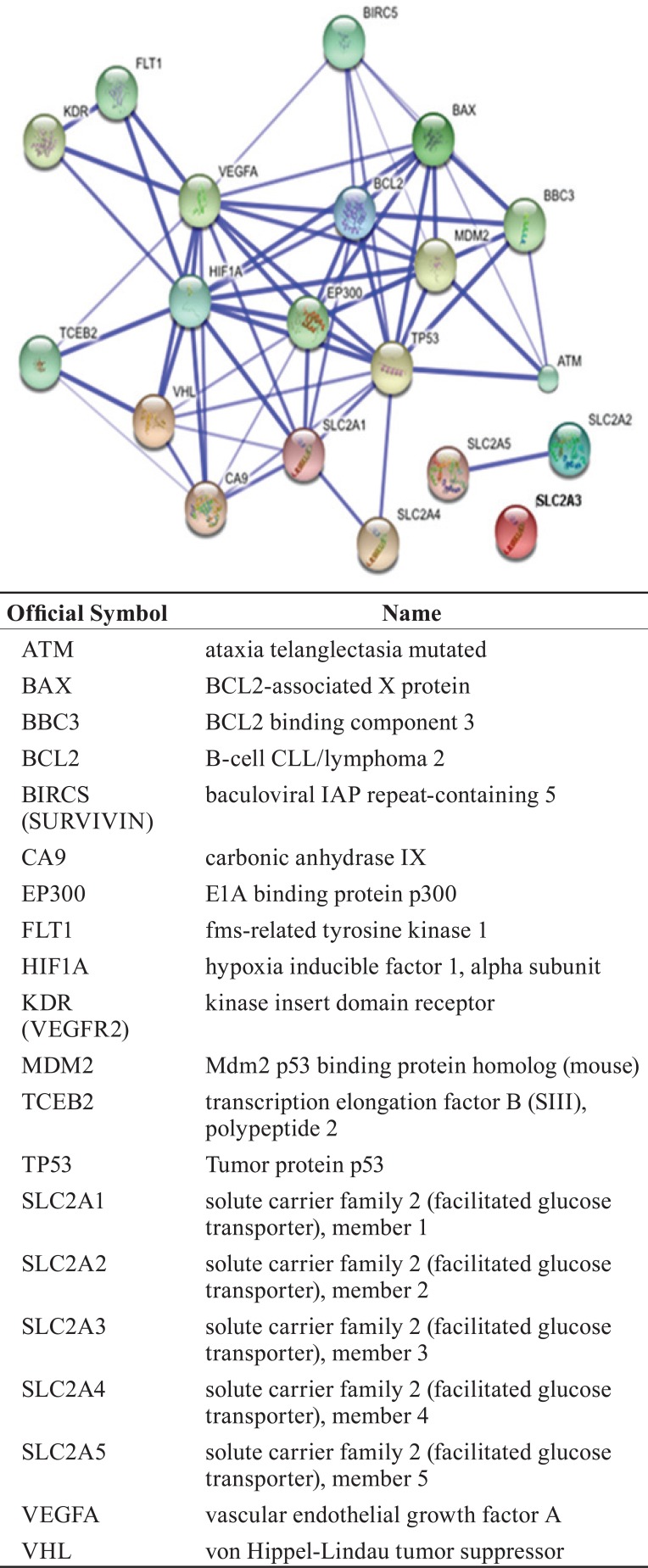
This is the confidence view. Stronger association are represented by thicker lines.

We used the STRING database to identify and predict interactions of the p53 protein with other proteins in the apoptosis, proapoptosis, angiogenesis and metabolic pathways. STRING assigns a confidence score to each predicted association (for more information visit the info section on the website http://string-db.org). The scores are derived from benchmarking the performance of the predictions against a common reference set of trusted, true associations. The results from a STRING search are visualised in Figure 1, and scores for predicted interactions are summarised in Table 1.

**Table 1:** The combined score between pair of proteins

Node 1	Node 2	Combined score	Node 1	Node 2	Combined score

GLUT1	VEGFA	0.842	HIF1α	EP300	0.999
TCEB2	VHL	0.999	VEGFA	VHL	0.978
ATM	BAX	0.507	GLUT1	TP53	0.800
KDR	FLT1	0.986	CA9	VEGFA	0.799
EP300	VHL	0.597	VEGFA	TCEB2	0.573
CA9	HIF1α	0.979	GLUT1	BCL2	0.800
VEGFA	TP53	0.955	VEGFA	FLT1	0.999
HIF1α	TCEB2	0.979	CA9	GLUT1	0.813
HIF1α	VHL	0.999	BCL2	MDM2	0.805
BCL2	EP300	0.983	BBC3	TP53	0.999
VEGFA	Survivin	0.562	VEGFA	HIF1α	0.999
TP53	MDM2	0.999	ATM	MDM2	0.990
BCL2	TP53	0.999	BBC3	BAX	0.996
HIF1A	BCL2	0.832	CA9	TCEB2	0.419
HIF1A	FLT1	0.995	BAX	MDM2	0.955
GLUT4	TP53	0.800	GLUT4	GLUT1	0.811
ATM	TP53	0.999	BBC3	MDM2	0.957
HIF1A	BAX	0.952	BBC3	BCL2	0.999
BBC3	Survivin	0.430	Survivin	BAX	0.627
VEGFA	EP300	0.962	Survivin	MDM2	0.430
CA9	TP53	0.629	BCL2	Survivin	0.727
VEGFA	BCL2	0.955	VEGFA	MDM2	0.901
CA9	EP300	0.543	BCL2	BAX	0.999
KDR	VEGFA	0.999	ATM	BBC3	0.688
CA9	VHL	0.807	GLUT5	GLUT2	0.912
BAX	EP300	0.949	TP53	VHL	0.690
VEGFA	BAX	0.740	GLUT1	HIF1α	0.997
GLUT2	VHL	0.571	EP300	MDM2	0.999
KDR	HIF1α	0.802	GLUT1	EP300	0.924
BAX	TP53	0.999	Survivin	TP53	0.736
HIF1α	TP53	0.999	TP53	EP300	0.999
HIF1α	MDM2	0.999	BBC3	EP300	0.489

The score is computed under the assumption of independence for the various sources in a naïve Bayesian fashion.

The database shows interactions that have association scores in the very high confidence range because only 8.5% of the data present had a score between 0.4 and 0.7, with the remaining scores above 0.7.

**Figure 2:**
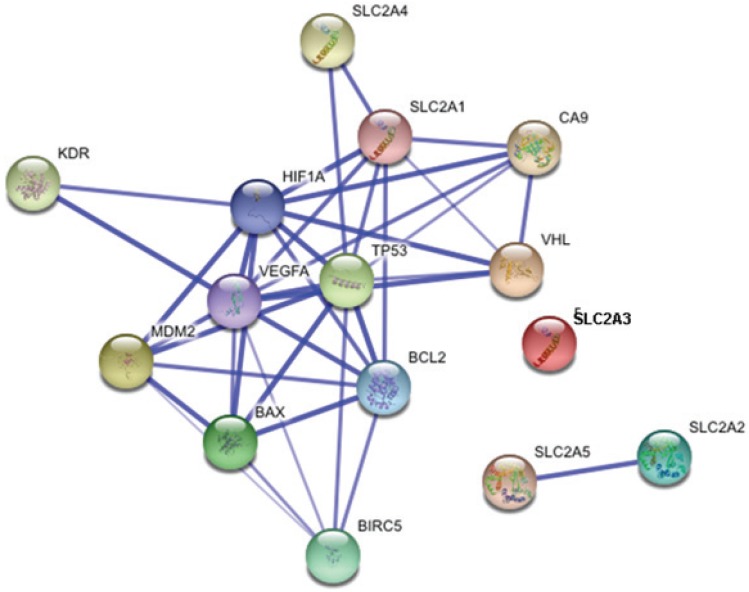
STRING interaction for proteins studied to find relevant networks in RCC. The score interaction is summarized in Table 1.

As a preliminary analysis, our group decided to study the proteins whose interactions may be viewed in detailed in Figure 2. The purpose of this analysis is to understand the interactions between these proteins in the samples of our patients that were previously predicted with STRING to have important interactions.

### Biostatistical analysis of molecular associations

Correlation between expression levels of chosen proteins are listed in Table 2.

**Table 2:** Biostatistical analysis of protein correlations using Pearson´s correlation coefficient test

Proteins	Glut1	Glut2	Glut3	Glut4	Glut5	Hif1-α	VEGF-A	VEGFR-2	CA9	VHL	BAX	MDM2	Survivin	Bcl-2	p53

Glut1		r=-0.045	r=0.256	r=0.209	r=0.359	r=0.122	r=0.199	r=0.044	r=0.362	r=-0.003	r=0.097	r=0.219	r=-0.088	r=-0.105	r=0.291
			*p*=0.022		*p*=0.001				*p*=0.001						*p*=0.009
Glut2	r=-0.045		r=-0.025	r=-0.154	r=-0.055	r=-0.032	r=0.127	r=0.067	r=0.097	r=0.041	r=0.008	r=0.006	r=-0.1	r=-0.051	r=0.087
Glut3	r=0.256	r=-0.025		r=-0.083	r=0.229	r=0.202	r=0.148	r=-0.187	r=0.270	r=0.053	r=-0.256	r=-0.002	r=-0.178	r=-0.123	r=0.090
	*p*=0.022				*p*=0.041				*p*=0.016		*p*=0.022				
Glut4	r=0.209	r=-0.154	r=-0.083		r=0.117	r=0.087	r=0.019	r=0.119	r=-0.042	r=-0.011	r=-0.1	r=0.194	r=0.169	r=-0.646	r=0.456
														*p*<0.01	*p*<0.01
Glut5	r=0.359	r=-0.055	r=0.229	r=0.117		r=0.520	r=0.229	r=-0.490	r=0.283	r=-0.028	r=-0.291	r=0.171	r=0.072	r=-0.646	r=0.103
	*p*=0.001		*p*=0.041			*p*<0.01	*p*=0.041	*p*<0.01	*p*=0.011		*p*=0.009			*p*<0.01	
Hif1-α	r=0.122	r=-0.032	r=0.202	r=0.087	r=0.520		r=0.225	r=-0.382	r=0.286	r=-0.131	r=-0.199	r=0.087	r=0.074	r=-0.665	r=0.061
					*p*<0.01		*p*=0.047	*p*=0.001	*p*=0.011					*p*<0.01	
VEGF-A	r=0.199	r=0.127	r=0.148	r=0.019	r=0.229	r=0.225		r=-0.009	r=-0.027	r=0.043	r=-0.111	r=-0.017	r=-0.083	r=-0.360	r=0.241
					*p*=0.041	*p*=0.047								*p*=0.001	*p*=0.031
VEGFR-2	r=0.044	r=-0.067	r=-0.187	r=0.119	r=-0.490	r=-0.382	r=-0.009		r=-0.119	r=0.263	r=0.182	r=0.042	r=-0.153	r=0.631	r=0.187
					*p*<0.01	*p*=0.001				*p*=0.018				*p*<0.001	
CA9	r=0.362	r=0.097	r=0.270	r=-0.042	r=0.283	r=0.286	r=-0.027	r=-0.119		r=0.176	r=0.171	r=0.243	r=-0.015	r=-0.160	r=0.083
	*p*=0.001		*p*=0.016		*p*=0.011	*p*=0.011						*p*=0.030			
VHL	r=-0.003	r=0.041	r=-0.053	r=-0.011	r=-0.028	r=-0.131	r=0.043	r=0.263	r=0.176		r=0.090	r=0.116	r=-0.119	r=0.254	r=-0.010
								*p*=0.018						*p*=0.023	
BAX	r=0.007	r=0.008	r=-0.256	r=-0.1	r=-0.291	r=-0.199	r=-0.111	r=0.182	r=0.171	r=0.090		r=-0.064	r=0.024	r=0.256	r=0.2
			*p*=0.022		*p*=0.009									*p*=0.022	
MDM2	r=0.219	r=0.006	r=-0.002	r=0.194	r=0.171	r=0.087	r=-0.017	r=0.042	r=0.243	r=0.116	r=-0.064		r=0.157	r=-0.080	r=0.091
									*p*=0.030						
Survivin	r=-0.088	r=-0.1	r=-0.178	r=0.169	r=0.072	r=0.074	r=-0.083	r=-0.153	r=-0.015	r=-0.119	r=0.024	r=0.157		r=-0.195	r=0.049
Bcl-2	r=-0.105	r=-0.051	r=-0.123	r=-0.646	r=-0.646	r=-0.665	r=-0.360	r=0.631	r=-0.106	r=0.254	r=0.256	r=-0.080	r=-0.195		r=-0.075
				*p*<0.01	*p*<0.01	*p*<0.01	*p*=0.001	*p*<0.01		*p*=0.023	*p*=0.022				
p53	r=0.291	r=0.087	r=0.090	r=0.456	r=0.103	r=0.061	r=0.241	r=0.187	r=0.083	r=-0.010	r=0.2	r=0.091	r=0.049	r=-0.075	
	*p*=0.009			*p*<0.01			*p*=0.031								

Among the associations, those found between p53 and Glut1, Glut4 and VEGFA (Pearson correlation coefficient r=0.291, significance level 0.009; r=0.456, significance level <0.01; and r=0.241, significance level 0.031, respectively) should be emphasised. An analysis using the Pearson’s correlation coefficient measured the strength of the linear relationship between p53 and proteins involved in the metabolic and angiogenic pathways. In the case of Glut1 and VEGFA, the r values were close to 0.3, suggesting some association between them. For Glut 4, we found a weak positive association, with r close to 0.5.

Another apoptosis regulator, BCL2, showed a strong negative linear correlation (r close to -0.7) with proteins involved in the metabolic and angiogenic pathways: Glut4, Glut5, HIF1α and VEGF-A. Negative values indicate a relationship between BCL2 and these proteins; as the values for BCL2 increase, the values for these proteins decrease. BCL2 also showed a strong positive association with KDR (VEGFR-2), with an r value close to 0.7.

### Relevance Networks

**Figure 3:**
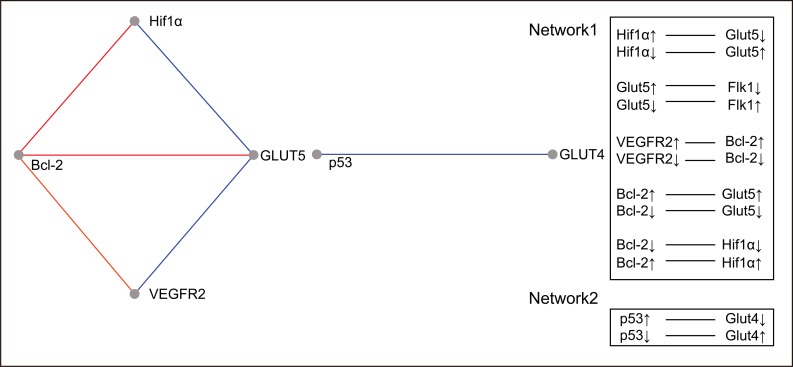
Relevance networks constructed. Proteins are represented as nodes in a network and edges are drawn between thm if their correlation coefficient falls between the minimum (r^2^=0.97) and maximum (r^2^=1) thresholds specified in the MeV module. Features without an association at ± 0.97 were removed. Links colored in red represent elements that are positively correlated while links colored in blue represent elements that are negatively correlated.

**Figure 4:**
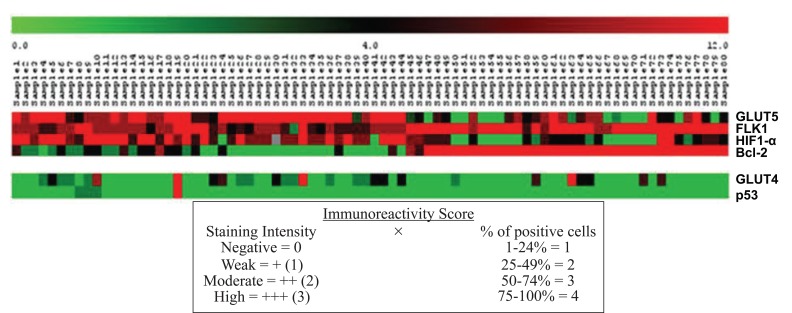
Subnets generated by the MeV module containing information about the IHC score expression of the 80 RCC samples regarding the networks predicted.

A relevance network (26) is a group of proteins whose expression profiles are highly predictive of one another. Using a permutation test, we evaluated the similarity of features by comprehensively comparing all features with each other in a pair-wise manner for the same cases. The correlation coefficients between proteins were calculated using the MeV module by comparing the expression pattern of each protein to that of every other protein. The ability of each protein to predict the expression of another protein was judged by the correlation coefficient. All features were connected to all other features with an absolute squared correlation coefficient (r^2^). We hypothesise that features with a high absolute (r^2^) represent hypotheses of a biological relationship. The program displays only the fraction of relationships at or above the chosen threshold, r^2^=1. Groups of features that are connected to each other with an absolute r^2^ higher than the threshold will aggregate and form a cluster or a relevance network. Figure 3 and Figure 4 show the relevance network and cluster results of the analysis of 15 markers in 80 cases of RCC.

Bioinformatics analysis leads us to 2 protein relevant networks. The first network consists of proteins involved in the angiogenesis pathway and the apoptotic suppressor, BCL2, and includes both the positive and negative correlations. The second network shows a negative interaction between the p53 tumour suppressor protein and the glucose transporter type 4.

## DISCUSSION

Recent advances in systems biology have enabled to create a cell-wide map of complex molecular interactions with the aid of the literature-based knowledgebase of molecular pathways (27). Relevant networks display nodes with varying degrees of cross-connectivity. An example of this can be observed in the networks in Figures 1 and 2, where different proteins actively involved in cancer and disease progression (cell cycle phase transitions, apoptosis, and cell migration) are highly cross-connected. They could represent the most trusted associations. Regulatory networks in eukaryotic cells hold complex interactions, however, because numerous post-translational interactions are involved in all critical biological functions.

To verify the *in silico* observations of the p53 protein network interaction *in vivo*, we conducted IHC studies in our cohort of RCC samples of 15 biomarkers contributing to renal carcinogenesis that can be divided into three categories by functional annotation. For proteins involved in the angiogenesis pathway, rapid tumour cell growth creates intracellular hypoxia, which initiates a series of cell signalling events that promote angiogenesis. Therefore, proteins that respond to changing intracellular oxygen concentration are likely to play critical roles in renal carcinogenesis. Among the significant proteins identified based on the STRING network (Figure 1), 11 belonged to the angiogenesis pathway or interacted with it, and 8 proteins (CA9, *SLC2A1* (Glut1), *SLC2A4* (Glut4), *SLC2A5* (Glut5), HIF1α, VEGFA, VEGFR2 and VHL) were shown to have significant associations with proteins belonging to the angiogenesis, apoptosis, or metabolic pathways in the 80 RCC studied. Proteins involved in the apoptosis pathway regulate the balance between cell proliferation and apoptosis and are influenced by genes that contribute to the development of cancer (oncogenes) and those that encode proteins that normally suppress tumour formation (tumour suppressor genes). A hallmark of cancer is the acquired resistance to programmed cell death or apoptosis. Therefore, proteins annotated with cell survival might be important in carcinogenesis. Of the 7 significant proteins identified by the STRING database of interacting proteins related to the apoptosis pathway, 4 showed significant correlations in the tumours studied: BAX, BCL2, MDM2 and p53. For proteins involved in the metabolic pathway, alterations in metabolism can have fundamental effects on almost every aspect of cell behaviour, including the ability to regulate proliferation, growth, and survival under conditions of variable nutrient and oxygen availability (28). The five significant proteins identified based on the STRING network, SLC2A1 (Glut1), SLC2A2 (Glut2), SLC2A3 (Glut3), SLC2A4 (Glut4), and SLC2A5 (Glut5), were annotated as having a meaningful role in our RCC cohort except for Glut2 (24).

Bioinformatics and biostatistical analysis of the IHC scores obtained from 15 markers studied in samples of patients affected by renal tumours seems to suggest the association of proteins related to different functional aspects in the tumour cell.

Because the study design emerged from our interest in understanding the p53 protein in the renal tumours under study, the associations found for this marker should be emphasised. Our results revealed a significant interaction between the p53 inducer of apoptosis and Glut1, Glut4 and VEGFA. The regulation of metabolic pathways is an important facet of p53 function that may provide some novel and effective therapeutic targets for cancer. It is almost impossible to address the metabolic changes in cancer without reference to the Warburg effect. The Warburg effect is the observation that most cancer cells predominantly produce energy by a high rate of glycolysis followed by lactic acid fermentation in the cytosol, in contrast to the comparatively low rate of glycolysis followed by oxidation of pyruvate in the mitochondria of most normal cells (29). The hypoxia inducible factor (HIF) directly activates the expression of most glycolytic enzymes. This fate may provide a partial explanation for the increased need for glycolysis-derived ATP because glycolysis is an oxygen-independent mechanism (28). Functions of p53 that can contribute to the dampening of glycolysis include the downregulation of expression of several glucose transporters (28). Bioinformatics analysis of RCC samples by the MeV tool revealed a relevant network between Glut4 and p53 that has a negative correlation. The trend in our samples is little detection of wild type p53, which could lead to increased Glut4 protein expression because transcriptional repression would only be very slight. One explanation for this finding is the knowledge that the inhibitory effect of p53 on the transcriptional activity of the GLUT4 promoter is significantly greater than its effect on the GLUT1 promoter. This may be due to the fact that Glut1 is a general “housekeeping” glucose transporter, whereas Glut4 is a tissue-specific and insulin-sensitive glucose transporter (30).

Tumours cannot grow beyond 2 to 3 mm without an adequate vascular supply. For this reason, tumours tend to recruit new blood vessels from the pre-existing vasculature by neoangiogenesis (31), and there is complexity and a richness of tumour vasculature in RCC. One of the most important drivers of metabolic reprogramming in a cancer cell is the response to hypoxia. Interestingly, hypoxia has also been shown to activate p53, although the mechanisms involved in this are not yet clear. Using a biostatistics test approach, we showed that p53 and VEGFA (a HIF1α transcriptional target) have a significant positive correlation in RCC samples. Under normoxic conditions, both p53 and HIF1α are low due to proteosome-mediated degradation. Mild or moderate hypoxia activates HIF1α-dependent angiogenesis, but it is not stringent enough to induce p53 accumulation. Under severe hypoxic conditions, p53 also accumulates, and when it reaches a threshold level, it binds to the oxygen dependent domain of HIF1α (32). Research on the relationship between p53 adaptation to low oxygen levels will help us understand this pathway and will provide new strategies for anti-tumour therapy.

One limitation of this work is that the analysis was restricted to only 15 biomarkers. This study is a preliminary trial in which we have studied a small number of biomarkers associated with the signalling pathways that are responsible for cellular processes, including proliferation, differentiation, apoptosis, and metastasis.

A second limitation of the analysis is that the approach to the expression of selected markers by means of TMA and IHC has not been combined with molecular biology techniques as (for example microarrays, polymerase chain reaction and western blotting). TMA has many advantages: (1) sections from TMA blocks can be utilised for the simultaneous analysis of up to 1,000 different tumours at the DNA, RNA or protein level; (2) TMA is highly representative of donor tissues; (3) TMA can improve the conservation of tissue resources and experimental reagents, improve internal experimental controls, increase sample numbers per experiment, and can be used for large-scale, massively parallel in situ analysis; and (4) TMA facilitates the rapid translation of molecular discoveries to clinical applications. IHC is the most routinely practiced technique for various analyses of histological samples including tumour identification. A high variability in staining is observed in intra-laboratory and inter-laboratory tests. This may be due to differences in antigenic epitopes, batch to batch variability in antibodies, differences in staining procedures and differences in the observation and interpretation of the staining results. TMA overcomes these factors and promises highly reliable quality assurance for IHC.

We have used IHC of primary tumours from patients to demonstrate for the first time the relevant interactions involved in different pathways that regulate cell fate.

An important next step will be to discover new protein interactions that could be extracted from *in vitro* biological networks, such as additional p53-related targets upstream and downstream of p53 that can be identified and validated for future discovery of novel compounds that target p53 signalling pathways (33). From this perspective, this work provides a comparison of biological knowledge of molecular interactions with experimental data.
